# Mechanochemical Preparation and Self-Assembly of Protein:Dye Hybrids for White Luminescence

**DOI:** 10.1021/acsapm.1c00382

**Published:** 2021-09-03

**Authors:** Yusheng Yuan, Niclas Solin

**Affiliations:** Department of Physics, Chemistry, and Biology, Biomolecular and Organic Electronics, Linköping University, Linköping 581 83, Sweden

**Keywords:** mechanochemistry, functionalization, amyloid fibrils, luminescent dyes, white light, FRET, protein, LED

## Abstract

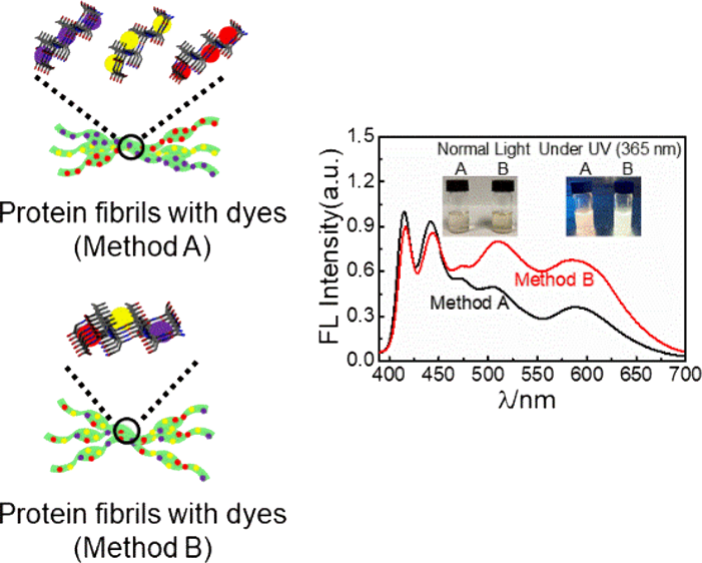

Protein nanofibrils (PNFs) functionalized with multiple dyes are prepared by a combination of mechanochemistry and liquid-phase self-assembly. The three employed dyes are Fluorescent Brightener 378 (F378), 2-butyl-6-(butylamino)-1*H*-benzo[*de*]isoquinoline-1,3(2*H*)-dione (Fluorol 555), and Nile red (NR). F378 acts as the donor with Fluorol 555 as the acceptor. F555 in turn acts as the donor and NR as the acceptor. This enables a FRET cascade that enables conversion of UV light to white light. The efficiency of FRET can be influenced by the details of the self-assembly process. If proteins milled with different dyes are mixed prior to self-assembly, nanofibrils are formed containing all three dyes, thus favoring FRET processes. By tuning the ratio of the three luminescent dyes, PNF dispersions are obtained that display bright white light emission. Moreover, the PNF dispersions can be converted into white luminescent films and gels where the PNFs may help to organize dye molecules. Additionally, the PNF materials can be employed as coatings on commercial LEDs, enabling emission of white light.

## 1 Introduction

With the increasing interest in the development of polymeric materials from sustainable sources, there has been renewed interest in employing bio-based polymeric materials for novel applications. For example, there has been much interest recently in developing luminescent materials based on biopolymers, and such materials have been applied to a wide variety of research areas including bioimaging,^1^ information encoding or encryption,^2^ biomimetic actuators,^3^ and LEDs,^4−6^ and a wide range of approaches are employed to generate white light.^7−9^ A wide range of biopolymeric materials have been employed to form luminescent materials, including DNA,^10,11^ fluorescent proteins,^12−14^ cellulose,^15,16^ and polysaccharides.^17,18^ These biopolymers possess several attractive characteristics. For example, DNA contains binding sites, including major and minor grooves, to which chromophores may bind, enabling functionalization. However, a drawback is that DNA is relatively sensitive to mechanical forces. On the other hand, cellulose nanofibrils have attractive mechanical properties but do not have the wide range of binding sites possessed by DNA, which makes functionalization with dyes more challenging even though cellulose can be stained by dyes as well as chemically modified enabling binding of emitters.^19^

The amyloid fibril is a type of biopolymeric material that combines the attractive features of DNA (structural features such as grooves where dyes may bind) and nanocellulose (mechanical strength) outlined above. Amyloid fibrils have traditionally been associated with diseases such as Alzheimer’s and Parkinson’s; however, in recent years, many examples of these so-called functional amyloids where amyloid structures have a functional value for the organism have been demonstrated.^20^ In addition, it has been demonstrated that a range of proteins not associated with diseases, including proteins from industrial waste streams,^21^ can form amyloid-like fibrils in vitro, typically by heating an acidic solution of the protein.^22,23^ Hereafter, we label such amyloid-like fibrils as protein nanofibrils (PNFs). PNFs have some common structural features, irrespective of the specific protein from which they are derived. PNFs are unbranched high aspect ratio particles with lengths in the micrometer range and widths of around 5–10 nm.^24^ Also at the molecular level, PNFs from different proteins share structural similarities. PNFs are typically bundles of highly ordered filaments composed of β strands that run perpendicular to the long fibril axis and results in arrays of β sheets.^25,26^ The mechanical properties of different PNFs have been investigated, and typical Young’s moduli of amyloid fibrillar materials range from 0.2 to 20 GPa.^27,28^ The preparation of PNFs in acidic water will result in an electrostatically stabilized colloidal PNF dispersion, which is convenient from a materials science perspective as this simplifies further processing. PNFs are investigated regarding a wide range of applications including tissue engineering,^29,30^ drug delivery.^31,32^ catalysis,^33^ and actuators.^34^

Due to the attractive structural features of PNFs (binding sites in combination with favorable mechanical properties), it is of interest to further investigate the use of PNFs as a template for organization of light-emitting dyes. Such functionalized PNFs can then be assembled into a variety of forms, including processable liquid dispersions, gels, and solid films, where the PNFs may help to organize dye molecules. As the majority of readily available dyes are highly hydrophobic and thus insoluble in water, which is the preferred solvent for the protein, it is of interest to develop methodology where hydrophobic dyes can be mixed with proteins. We have previously demonstrated that mechanochemical methodology can be employed. A protein capable of self-assembly into PNFs is ground with a hydrophobic luminescent dye, and the resulting hybrid material is then dissolved/dispersed in water. Upon heating in acidic water, PNFs are formed, which are functionalized by the luminescent dyes. These functionalized PNFs can be used in organic electronic applications^35−37^ and even control the photophysical properties of dyes.^38,39^ The mechanochemical functionalization of PNFs with luminescent dyes constitutes a valorization process that may enable novel applications for PNF materials. It is of interest to investigate if the prepared materials can be employed for applications involving donor–acceptor pairs of dyes. There has been much interest recently in mimicking natural light-harvesting processes by achieving efficient energy transfer from donors to acceptors through the Förster resonance energy transfer (FRET) process.^40−42^ Such an approach can moreover be employed to achieve systems capable of emission of white light.

Hen egg white lysozyme (HEWL) is an interesting protein source for PNFs as it is readily converted into PNFs by heating an acidic aqueous solution of it. Moreover, HEWL is readily available in large quantities, and HEWL finds use in food preservatives (with E number E1105). HEWL can thus be a relatively low-cost source of protein nanomaterials.

Herein, we demonstrate that HEWL–protein nanofibrils (LPNFs) functionalized with multiple dyes can be utilized as a FRET system capable of converting UV light to white light. We moreover demonstrate that the FRET process can be tuned by slight modifications during the PNF formation process. We demonstrate two different approaches to convert the PNF dispersions into solid materials: (i) spray coating of the LPNF dispersion, resulting in thin solid LPNF films; (ii) and gelation by mixing the LPNF dispersion with polyvinyl alcohol (PVA) in glycerol (Gly)/H_2_O binary solution, resulting in moldable gels. In addition, these moldable gels can be used to coat commercial UV-LEDs, enabling conversion of UV light to white light.

## 2. Materials and Methods

### 2.1 Materials

4,4′-Bis(2-methoxystyryl) biphenyl (Fluorescent Brightener 378, F378) was obtained from TCI. (2-Butyl-6-(butylamino)-1*H*-benzo[*de*]isoquinoline-1,3(2*H*)-dione) (Fluorol 555, F555) was obtained from Exciton (Dayton, OH). Nile red, hen egg white lysozyme (HEWL), poly(vinyl alcohol) (PVA, *M*_W_ 115,000), glycerol (99%), coumarin 153, fluorescein, and concentrated HCl were purchased from Sigma-Aldrich. All chemicals were used as received without further purification, and doubly distilled water (18.2 Ω) was used throughout.

### 2.2 Characterization

Absorption spectra were determined on a UV-2450 spectrophotometer (Shimadzu, Japan). Samples measured were diluted 10 times with 25 mM HCl from the reaction mixture. Fluorescence spectra were collected using a Horiba Jobin-Yvon Fluoromax-4 spectrometer using a 1 cm path length. Prior to measurement, samples were diluted 20 times with 25 mM HCl from the reaction mixture. Atomic force microscopy (AFM) measurements were obtained using a digital instruments dimension 3100 atomic force microscope. The initial reaction mixtures were diluted 100 times prior to applying to silica substrates and left to dry for 1 min. Excess fluid was removed by applying a nitrogen gas flow. The fluorescence microscope images were recorded with an epifluorescence microscope (Zeiss Axiovert inverted microscope A200 Mot) equipped with a CCD camera (Axiocam HR), along with a microscope illuminator (HBO100, mercury vapor short-arc lamp). A biotrode pH meter (Hamilton Bonaduz AG, Switzerland) was used for pH measurements. The time-correlated single-photon counting (TCSPC) measurements were performed on an Edinburgh Instruments spectrometer (FLS 1000) with a 375 nm pulsed laser. A scanning electron microscope (Zeiss Leo 1550, Germany) with an acceleration voltage of 5 kV was used to get the SEM images after painting Pt on the film for 10 s.

### 2.3 Materials Preparation

#### 2.3.1 Preparation of Functionalized PNFs

First, 50 mg of HEWL is ground separately with either F378 (0.5 mg), F555 (0.5 mg), or NR (0.5 mg) for 10 min with a mortar and pestle. The resulting powder is then dissolved in 5 mL of 25 mM hydrochloric acid followed by filtration through 0.45 μm PES filters. This results in three solutions of protein with their respective dye, labeled F378@HEWL, F555@HEWL, and NR@HEWL. The dyes@LPNF hybrids were then prepared by two different methods. In the first method (labeled method A in the main text), the three resulting dispersions are each heated at 80 °C with magnetic stirring at 1000 rpm for 24 h, to obtain F378@LPNF, F555@LPNF, and NR@LPNF, respectively. These three LPNF dispersions are then mixed, resulting in a material labeled F378@LPNF/F555@LPNF/NR@LPNF. In the other method, labeled method B in the main text, F378@HEWL, F555@HEWL, and NR@HEWL are mixed prior to PNF formation. The resulting mixture is then heated at 80 °C with magnetic stirring at 1000 rpm for 24 h to obtain multiple functionalized LPNFs labeled F378:F555:NR@LPNF.

#### 2.3.2. Preparation PNF Films by Spraying

The F378:F555:NR@LPNF dispersion was transferred to a spray bottle and was then sprayed onto a glass substrate (size 2 × 2 cm, procedure is shown in Figure S1), and the resulting film was allowed to dry. Upon exposure to UV light (365 nm), the film displayed white luminescence.

#### 2.3.3. Preparation of Gels and LED Coatings

The white-gel coatings were prepared as follows: 1 g of PVA was first dissolved in a mixture of glycerol (Gly) and water (20 mL, Gly:H_2_O, 3:2) at 90 °C. After entirely being dissolved, the homogeneous solution was then mixed with the F378:F555:NR@LPNF dispersion under stirring (1:1, v/v). The final mixture was transferred into a suitable mold. In the case of formation of an LED coating, a tube-formed mold was employed and a commercial LED (LED supply, 5 mm in diameter and projects light at a half angle of 15°) was inserted into the middle of the gelation agent; the mixture was then held at room temperature and kept under ambient conditions overnight, resulting in formation of a curved gel that could be readily separated from the mold. White emission was obtained by systematically varying dye concentrations and validated using CIE 1931 chromaticity coordinates.

#### 2.3.4 CRI and Luminescence Characterization of White Devices on UV-LED

For long-term stability tests, the devices were operated at 3.5 V under ambient conditions for 4 days. For measurements, the LED was transferred to a glove box equipped with a QE Pro spectrometer (Ocean Optics) with an integrating sphere and a Keithley 2400. Luminescence spectra as well as CRI and luminance were monitored for 15 min at a driving voltage of 3.5 V. The characterization of spray-coated films was done as follows: a spray-coated film (on a glass substrate) was placed above a 365 nm LED at a distance of 2 cm. During measurements, in order to obtain a well-defined area, a mask with an area of 0.073 cm^2^ was employed. For gels coated directly onto LEDs, the white luminescent gel was directly put onto a 365 nm LED, and the device was then characterized as described above. The temperature of the gel device on the LED was monitored using a thermographic camera 225s (FOTRIC). The luminous efficacy (η, lm·W^–1^) was calculated through the luminous flux divided by the input electric power.^43^ The correlated color temperature (CCT) was calculated by the equation^44^
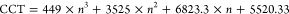

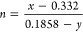
where *x* and *y* correspond to CIE data.

## 3. Results and Discussion

### 3.1 Mechanochemical Preparation of Functionalized LPNFs

**Figure 1:**
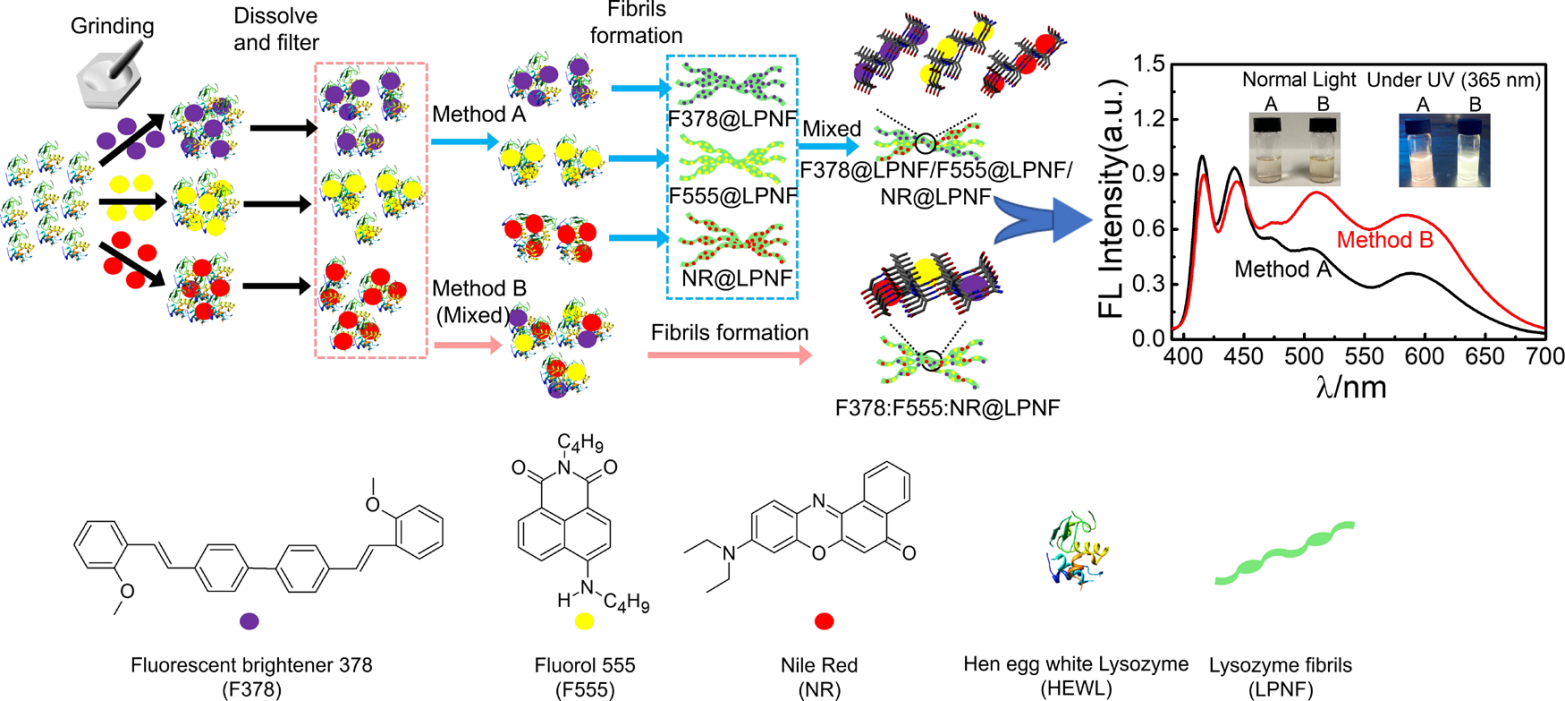
Schematic illustration of material preparation and self-assembly of proteins functionalized with dyes into PNFs by two different methods A and B. Fluorescence spectra obtained by excitation at 365 nm are shown to the right with the insets being photographs of PNF dispersions prepared by method A or B, under normal light and UV light.

Hen egg white lysozyme (HEWL) is milled separately with three different dyes (Fluorescent Brightener 378 (F378), 2-butyl-6-(butylamino)-1*H*-benzo[*de*]isoquinoline-1,3(2*H*)-dione (F555), and Nile red (NR), see Figure 1 for chemical structures) that emit light in the blue, green, and red parts of the visible spectrum, respectively. The resulting hybrids (F378@HEWL, F555@HEWL, and NR@HEWL) are then dissolved in acidic water, and LPNFs are formed by two different methods (Figure 1). In method A, each material (HEWL milled with one dye) is heated separately, resulting in an LPNF functionalized with one type of dye. The resulting three types of PNFs are hereafter labeled F378@LPNF, F555@LPNF, and NR@LPNF. The three different LPNF preparations are then mixed, resulting in an LPNF dispersion containing fibrils with all three dyes but where individual fibrils contain only one type of dye (method A). This type of mixture, formed by method A, is hereafter labeled F378@LPNF/F555@LPNF/NR@LPNF. On the other hand, in method B, the three preparations (F378@HEWL, F555@HEWL, and NR@HEWL) are mixed before heating, resulting in a solution of protein and all three dyes. When this mixture is heated, PNFs will form that contain a statistical mixture of all three dyes (method B). This type of mixture, formed by method B, is hereafter labeled F378:F555:NR@LPNF. In preliminary tests, it was found that the ratio of dyes could be adjusted so that white light was generated when samples were excited at 365 nm. It was moreover found that for identical dye ratios, LPNFs prepared by method B showed more efficient FRET compared to LPNFs prepared by method A.

### 3.2 Structural Characterization of Functionalized LPNFs

**Figure 2:**
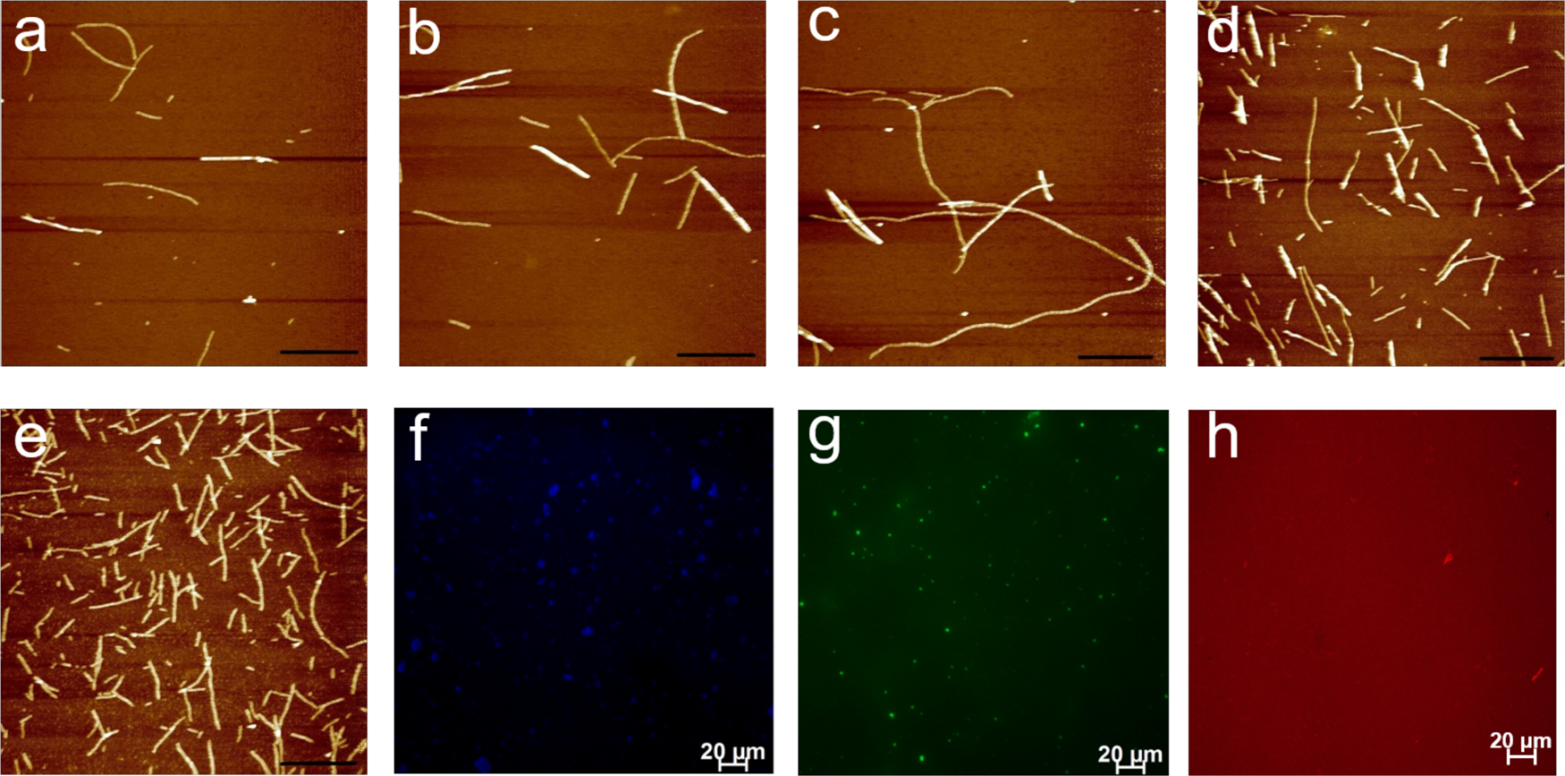
AFM images of functionalized protein fibrils. (a) F378@LPNF, (b) F555@LPNF, (c) NR@LPNF, (d) F378@LPNF/ F555@LPNF/NR@LPNF (prepared by method A), and (e) F378: F555: NR@LPNF (prepared by method B). Scale bars represent 2 μm. (f–h) Fluorescence microscope images of (f) F378@LPNF, (g) F555@LPNF, and (h) NR@LPNF. Scale bars represent 20 μm.

Functionalized LPNFs were prepared according to method A or B, and the morphology of the various types of LPNFs was investigated by atomic force microscopy (AFM). Figure 2a–c shows AFM images of LPNFs functionalized with one type of dye. In all cases, LPNFs can readily be observed. Figure 2d shows an AFM image of a sample after the three types of LPNFs (of the type shown in Figure 2a–c) were mixed into one sample (F378@LPNF/F555@LPNF/NR@LPNF), corresponding to method A. Figure 2e shows LPNFs prepared according to method B, resulting in LPNFs functionalized with the three dyes F378, F555, and NR (F378:F555:NR@LPNF). These results clearly indicate that lysozyme proteins milled with various dyes are still capable of forming LPNFs in agreement with previous studies.^45^

The F378@LPNF, F555@LPNF, and NR@LPNF materials were further characterized by fluorescence microscopy. The employed epifluorescence microscope allows excitation to be performed at 430, 488, and 546 nm; accordingly, drop-casted samples of F378@LPNF (excited at 430 nm), F555@LPNF (excited at 488 nm), and NR@LPNF (excited at 546 nm) were excited at the wavelength closest to the absorption maximum of the dye. In the fluorescence microscope images (Figure 2f–h), the different colors obtained from emission of the different dyes are clearly discernible. It should be noted that LPNFs display intrinsic fluorescence^46^ that, however, is weak compared to the emission from F378.

### 3.3 Optical Properties of Dyes and Functionalized LPNFs

**Figure 3:**
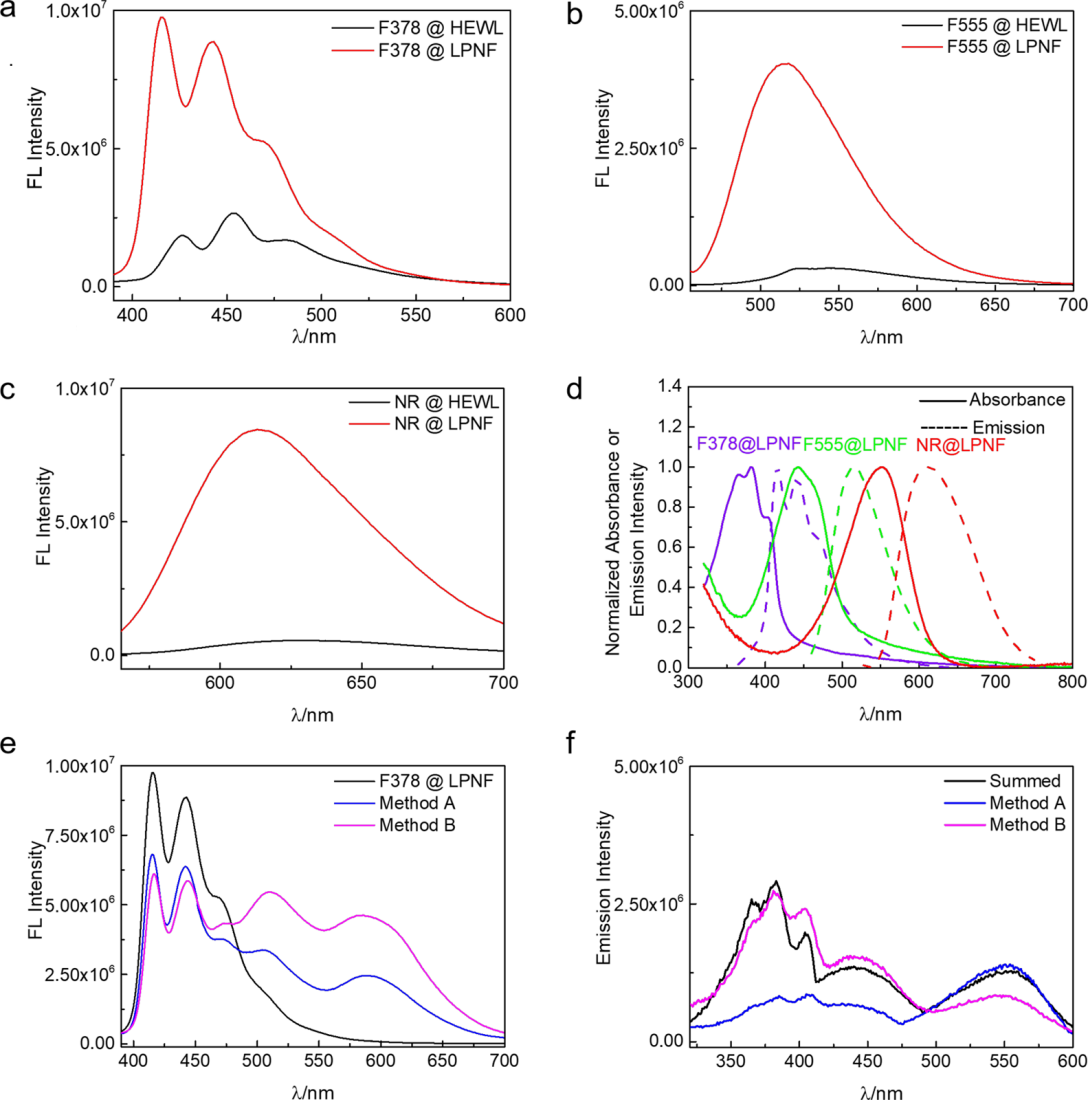
(a–c) Fluorescence spectra of LPNFs functionalized with individual dyes. (a) F378@LPNF, excited at 365 nm; (b) F555@LPNF, excited at 440 nm; and (c) NR@LPNF, excited at 550 nm. (d) Overlap of absorbance (solid lines) and emission (dashed lines) of F378, F555, and NR in the protein fibril matrix. The absorbance and emission intensities were normalized to 1 at their maximum value. (excitation, λ = 365, 440, and 550 nm separately). (e) LPNFs functionalized with dyes in different processes excited at 365 nm. (black line: F378@LPNF, blue line: F378@LPNF/ F555@LPNF/NR@LPNF in process A, and pink line: F378:F555:NR@LPNF in process B). (f) Excitation spectra were obtained by monitoring the emission of NR at 610 nm. The summed excitation spectrum was obtained by monitoring the emission of F378 at 420 nm, F555 at 520 nm, and NR at 610 nm. [F378]: 0.25 μg·mL^–1^, [F555]: 0.25 μg·mL^–1^, and [NR]: 30 ng·mL^–1^.

The fluorescence emission spectra were also determined for LPNFs functionalized with the individual dyes (F378@LPNF, F555@LPNF, and NR@LPNF). The corresponding absorbance spectra are shown in Figure S2 (Supporting Information). For reference, the fluorescence spectra of the corresponding proteins before fibrillation are included (F378@HEWL, F555@HEWL, and NR@HEWL). As shown in Figure 3a–c, the LPNFs doped with F378, F555, and NR separately display a huge increase in fluorescence intensity compared to the same materials before formation of LPNFs. The changes are typical of processes related to changes in the aggregation state of dyes where typically monomer states (where the dye is dispersed in a matrix) have a higher fluorescence quantum yield^38^ compared to the aggregated state. In order to ensure efficient FRET, an important parameter is the overlap between the donor’s emission spectrum and the acceptor’s absorbance spectrum. Figure 3d shows the plotted absorbance and emission spectra for samples of F378@LPNF, F555@LPNF, and NR@LPNF. Inspection of the respective spectra clearly shows that F378-F555, F378-NR, and F555-NR should be able to function as donor–acceptor pairs in FRET.

The optical properties of LPNFs functionalized with three dyes (by methods A and B) were characterized (Figure 3e) and compared to the spectra of LPNFs functionalized with individual dyes (Figure 3a–c). When F378@LPNF is excited at 365 nm, F378 shows strong fluorescence as the dye is directly excited. In order to verify that F555 and NR are not directly excited by 365 nm light, control experiments were performed where samples of F555@LPNF or NR@LPNF are excited at 365 nm (Figure S3a, Supporting Information); however, the fluorescence intensity is much lower than when the dyes are excited at their optimum wavelength (Figure 3a–c), meaning that direct excitation of F555 and NR plays a minor role in this system. Another potential FRET pair is between F378 and NR; as shown in Figure S3b, there is a possible energy transfer from F378 to NR, but the efficiency is quite low. Further samples were then investigated containing all three dyes. Both samples consisting of F378@LPNF/ F555@LPNF/NR@LPNF (prepared by method A) and F378:F555:NR@ LPNF prepared by method B were investigated. In the sample prepared by method A, there is a decrease in intensity of emission from F378 and a concomitant increase in fluorescence from F555 and NR. However, the sample prepared by method B shows a higher degree of fluorescence intensity from F555 and NR, meaning that the energy transfer is more efficient in the sample prepared by method B compared to method A. The same trend is apparent when the samples are investigated with regard to their excitation spectra. The excitation spectra were obtained by monitoring emission of NR at 610 nm. The sample prepared by method B shows a more significant contribution from F378 and F555 to the NR emission compared to the sample prepared by method A. These results combined indicate that FRET is occurring from the excited state of F378 to the ground state of F555 (F378 → F555) and then from the excited state of F555 to the ground state of NR (F555 → NR). The results in Figure 3e,f clearly shows that FRET is more efficient in samples prepared by method B compared to samples prepared by method A. The deconvolution of the fluorescence spectra obtained from samples prepared by methods A and B are plotted in Figure S4 (Supporting Information). In order to get efficient FRET, molecules in FRET systems should have the following prerequisites: (i) the distance between donors and acceptors should be positioned in proximity of each other (typically 1–10 nm)^47,48^ and (ii) the emission spectrum of the donor should overlap with the absorption spectrum of the acceptor.^49^ As the samples prepared by method B contain fibrils with a statistical distribution of all three dyes, more efficient FRET in samples prepared by method B is logical, as it would be expected that the average distance between donor–acceptor pairs will be shorter in samples prepared by method B compared to samples prepared by method A, where individual LPNF only contain one type of dye. In addition to hetero-FRET (involving a different donor and acceptor molecule), the possibility of homo-FRET (where the same type of molecule functions as a donor and acceptor) should be pointed out.

### 3.4 Optimization of Dye Composition for White Light Luminescence

**Figure 4:**
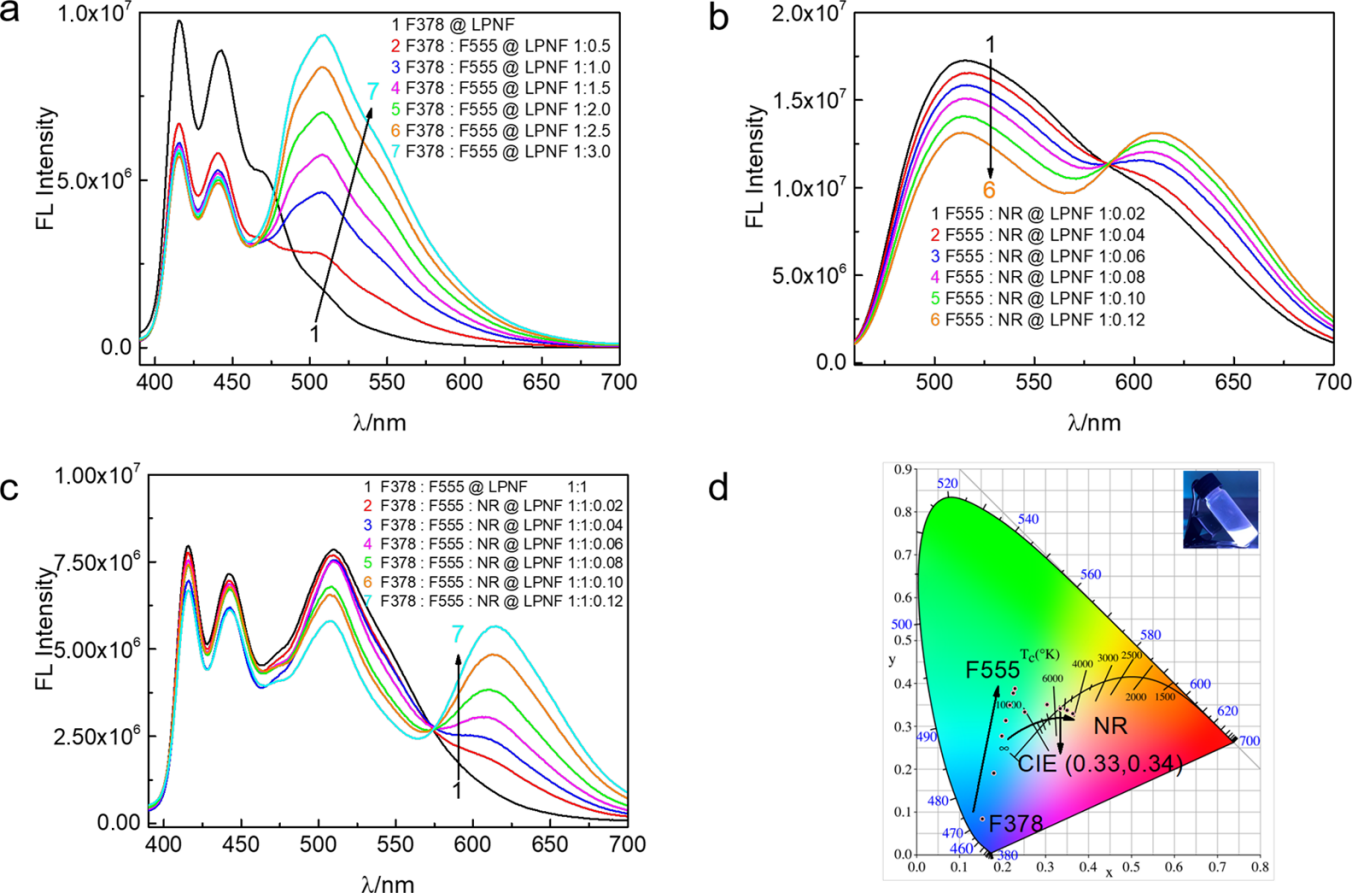
(a) Quenching of F378@LPNF emission (0.25 μg·mL^–1^) as a function of F555@LPNF concentration (0.125–0.75 μg·mL^–1^) in liquid state. (b) Quenching of F555@LPNF emission (0.25 μg·mL^–1^) as a function of NR@LPNF concentration (5–30 ng·mL^–1^) in liquid state. (c) Quenching of F378: F555@LPNF emission (0.25 μg·mL^–1^) as a function of NR@LPNF concentration (5–30 ng·mL^–1^) in liquid state. (d) Chromaticity data (CIE 1931) of the white light emitted by balancing the ratio of F378:F555:NR@LPNF in liquid state. The inset is a photograph of an F378:F555:NR@LPNF dispersion under illumination of UV light (365 nm).

In order to optimize the ratio of the dyes suitable for white light emission, further experiments were carried out to investigate the FRET process for samples prepared by method B. Initially, the process was investigated for samples only involving a single donor–acceptor pair. In the discussion below, the concentration of dyes in the protein used to form LPNFs is used. For example, Figure 4a shows the fluorescence spectra for samples obtained by keeping the concentration of F378@HEWL (0.25 μg·mL^–1^, Figure 4a) constant while increasing the concentration of F555@HEWL (0.125–0.75 μg·mL^–1^). After fibril formation, this will result in F378:F555@LPNF samples with a varying ratio between F378 and F555 dyes. As can be seen, when the amount of F555 relative to F378 is increased, the contribution of emission from F378 (420/445 nm) is diminished while the emission from F555 (520 nm) is gradually enhanced. In Figure 4b, the concentration of F555@HEWL (0.25 μg·mL^–1^) was fixed while increasing the concentration of NR@HEWL (5–30 ng·mL^–1^), resulting in formation of F555:NR@LPNF with varying ratio between F555 and NR. When the amount of NR relative to F555 is increased, the emission of F555 (520 nm) gradually decreased with a concomitant increase of the emission from NR. In addition, the FRET process involving all three dyes was investigated by keeping constant the concentration of F378: F555@HEWL while varying the concentration of NR@HEWL (Figure 4c), resulting in F378:F555:NR@LPNF with a constant concentration of F378 and F555 and a varying concentration of NR. Due to the energy transfer between F378 to F555 and F555 to NR, white luminescence can be achieved by increasing the concentration of NR.

As is well known in the art, white light is achieved by combining various colors of light generated from red, green, and blue (RGB) lights. In the field of colorimetry, colors are quantified by chromaticity coordinates, of which the most widely used are the CIE (Commission Internatio male de l’Eclairage) 1931 (*x*, *y*, and *L*) chromaticity coordinates. Here, the combination of *x* and *y* defines the color and *L* defines the brightness (luminosity).^50^ The emission colors in the chromaticity diagram (CIE1931) were calculated from the fluorescence spectra of the samples under 365 nm irradiation, and the results are shown in Figure 4d. The emission color could be successfully tuned from blue (0.15, 0.08) to white (0.33, 0.34) by using different ratios of F555 and NR (Tables S1–S3, Supporting Information). The coordinates are very close to the CIE coordinate (0.33, 0.33) of the pure white light.^51^

## 4. Förster Resonance Energy Transfer (FRET) Parameters of Functionalized LPNF

### 4.1 Fluorescence Decay in LPNF:Dye Hybrids

**Figure 5:**
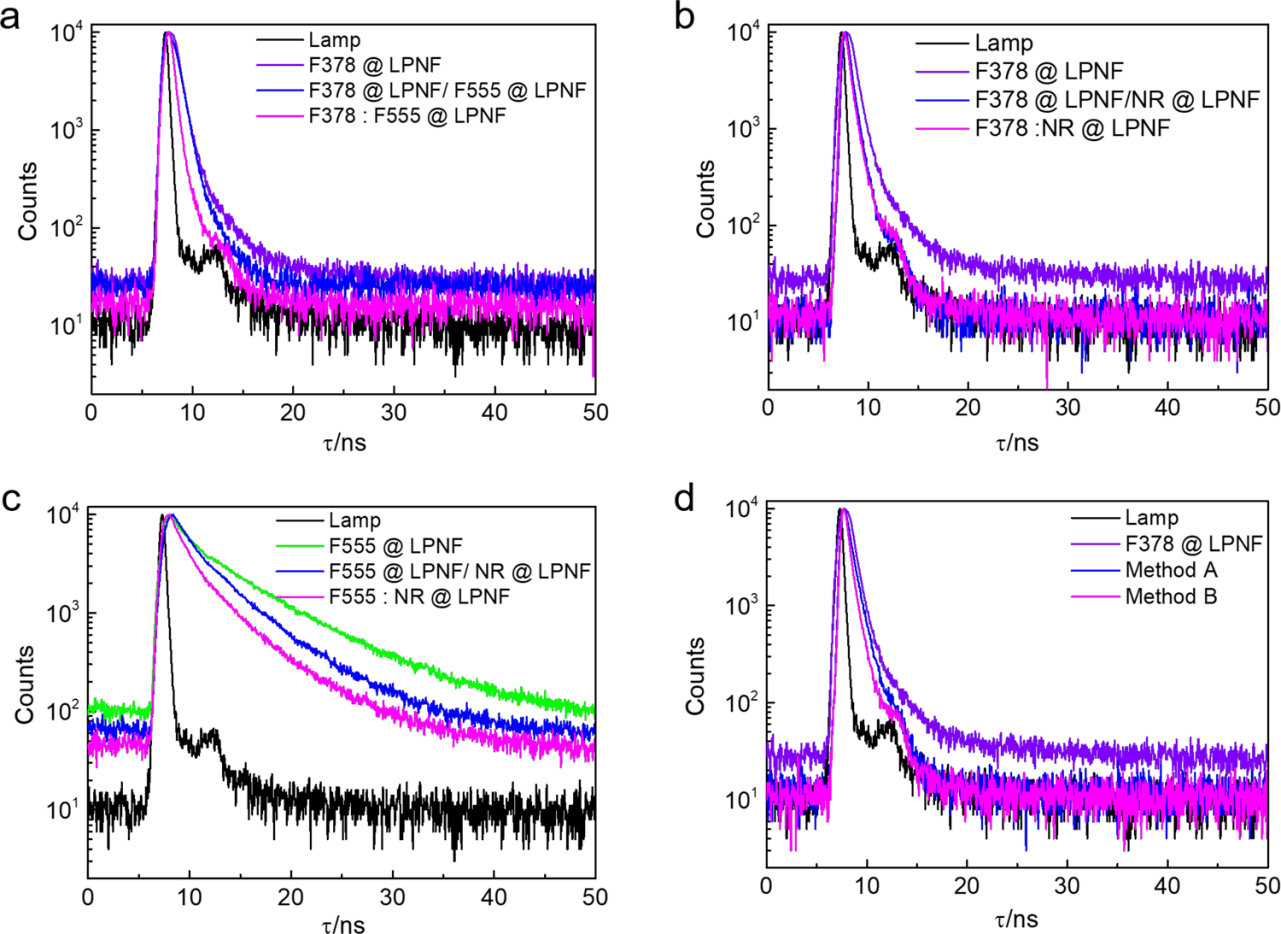
(a) Fluorescence decay profiles of LPNFs functionalized with F378 and F555 in different processes. Lamp (black line), F378@LPNF (violet line, monitored at 420 nm), F378@LPNF/F555@LPNF (blue line, monitored at 420 nm), and F378: F555@LPNF (magenta line, monitored at 420 nm). (b) Fluorescence decay profiles of LPNFs functionalized with F378 and NR in different processes. F378@LPNF/NR@LPNF (blue line, monitored at 420 nm) and F378:NR@LPNF (magenta line monitored at 420 nm). (c) Fluorescence decay profiles of LPNFs functionalized with F555 and NR in different processes. F555@LPNF (green line, monitored at 520 nm) and F555@ LPNF/ NR@LPNF (blue line, monitored at 520 nm). F555: NR@LPNF (magenta line, monitored at 520 nm). (d) Fluorescence decay profiles of various functionalized LPNFs: F378@LPNF (violet line, monitored at 420 nm) and F378@LPNF/F555@LPNF/NR@LPNF (method A, blue line, monitored at 420 nm). F378: F555: NR@LPNF (method B, magenta line, monitored at 420 nm). [F378]: 0.25 μg·mL^–1^, [F555]: 0.25 μg·mL^–1^, and [NR]: 30 ng·mL^–1^.

Fluorescence decays were obtained for samples of LPNFs functionalized with individual dyes as well as LPNFs functionalized with two or three dyes. For samples in the liquid state, the fluorescence decay was investigated for samples prepared by either method A or B. For gels and films, only the samples prepared by method B were investigated. When extracting excited state lifetimes from the decay profiles, it proved necessary to fit decay profiles as a triple exponential decay, in order to obtain reasonably low χ^2^ values (Table S4, Supporting Information). The lifetimes for donors@LPNF are 0.91 ns (F378@LPNF) and 6.80 ns (F555@LPNF), while NR@LPNF has a lifetime of 1.82 ns. When the lifetime of F378 is compared for samples prepared by methods A and B, there is a trend where the samples prepared by method A has a longer lifetime than the samples prepared by method B. In the case of F378@LPNF/F555@LPNF (method A), the lifetime is 0.56 ns, whereas for F378:F555@LPNF (method B), the lifetime is 0.52 ns (Figure 5a). When the lifetime of F555 is compared in the presence and absence of NR, a similar trend can be observed (Figure 5c). Whereas the lifetime for F555@LPNF is 6.80 ns, the lifetime is shortened to 4.63 nm for F555@LPNF/NR@LPNF (method A) and 4.07 ns for F555:NR@LPNF (method B). The lifetimes obtained from F378@LPNF/NR@LPNF (method A) and F378:NR@LPNF (method B) samples display only a minor difference of 0.85 and 0.84 ns, respectively (Figure 5b). Finally, the FRET in systems involving three dyes was investigated (by monitoring F378 decay at 420 nm) for samples prepared by either method A or B (Figure 5d). As mentioned above, the fluorescence lifetime of F378@LPNF is 0.91 ns, and for samples prepared by method A (F378@LPNF/F555@LPNF/NR@LPNF) and method B (F378:F555:NR@LPNF), the lifetime decreased to 0.83 and 0.77 ns, respectively. The F378 excited state lifetime for samples prepared by method B is accordingly shorter than for samples prepared by method A. Furthermore, the F378 lifetime of gels and sprayed films and gels (both containing F378:F555:NR@LPNFs prepared by method B) was 0.70 and 0.67 ns, respectively (Figure S5, Supporting Information). By comparing the results from liquid dispersion of PNFs prepared by method B and the same material converted into gels or solid films, it can be observed that the lifetime gets shorter in the latter cases. This is an indication that for these samples, the average distance between donor and acceptors are shorter than in the liquid dispersion.

### 4.2 Energy Transfer Efficiency of LPNF:Dye Hybrids

The efficiency of energy transfer (η) can be estimated from changes in lifetime of a donor in the absence (τ_D_) and presence (τ_DA_) of an acceptor be employing the eq η = 1 – τ_DA_/τ_D_. As shown in Table 1, for F378 and F555 samples, the η is 38.5% for method A and 42.9% for method B. In the case of F378 and NR samples, η is slightly increased from 6.6% in method A to 7.7% in method B. Similarly, for the F555 and NR samples, η increases from 31.9% in method A to 40.1% in method B. For the LPNF samples containing three dyes (F378/F555/NR), η increases almost by a factor of 2 in method B (15.4%) compared to method A (8.8%). For gels and solid samples (both prepared by method B), η is 23.1% for gel and 26.4% for white film.

**Table 1:** FRET Parameters of LPNFs Functionalized with Dyes^a^

samples	η %	*J*(λ) 10^15^ M^–1^ cm^–1^·nm^4^	τ (ns)	*R*_0_ (nm)	*R*_DA_ (nm)
F378@LPNF sol	*	0.26^#^	0.91	5.5^#^	*
F555@LPNF sol	*	0.34^#^	6.80	5.3^#^	*
NR@LPNF sol	*	0.51^#^	1.82	5.5^#^	*
F378@LPNF/F555@LPNF sol	38.5	3.0	0.56	8.2	8.9
F378: F555@LPNF sol	42.9	3.0	0.52	8.2	8.6
F378@LPNF/NR@LPNF sol	6.6	1.0	0.85	6.9	10.7
F378: NR@LPNF sol	7.7	1.0	0.84	6.9	10.4
F555@LPNF/NR@LPNF sol	31.9	3.3	4.63	7.8	8.8
F555: NR@LPNF sol	40.1	3.3	4.07	7.8	8.3
method A sol	8.8	*	0.83	*	*
method B sol	15.4	*	0.77	*	*
white gel (method B)	23.1	*	0.70	*	*
white film (method B)	26.4	*	0.67	*	*

a Sol is the solution state. Quantum yields for dyes@LPNFs: Φ_F378_: 0.72, Φ_F555_: 0.47, Φ_NR_: 0.38. [F378@LPNF]: 0.25 μg·mL^–1^, [F555@LPNF]: 0.25 μg·mL^–1^, and [NR@LPNF]: 30 ng·mL^–1^. * means not given. ^#^ is the value for homo-FRET.

### 4.3 FRET Parameters of LPNF:Dye Hybrids

This section focuses on FRET from F378 to one or two acceptors. The type of energy transfer can be determined from the Förster distance (*R*_0_) values. In order to estimate *R*_0_, the orientation factor κ^2^, taking into account the relative orientation of donor and acceptor needs to be known. In general, anisotropic dyes will bind with their long axis parallel to the long PNF axis.^52−54^ However, as we do not have access to detailed information of the relative orientation of dyes for the samples investigated herein, we assume an isotropic orientation as a rough approximation. In addition, the quantum yields of the donor molecules (F378 and F555) are required. We have accordingly estimated the quantum yield for PNFs functionalized with one dye using either coumarin 153 (C153) (for F378 and F555) or fluorescein as standards (for NR). The quantum yield of F378@LPNF, F555@LPNF, and NR@LPNF samples are 0.72, 0.47, and 0.38, respectively (Figure S6, Supporting Information), in agreement with earlier reported results for these molecules in various solvents and matrices.^55−57^

The following equations can be used to calculate the *R*_0_ values in isotropic media^58^
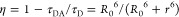
1
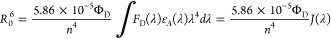
2where *n*, *F*_D_ (λ), and ε*_A_*(λ) are the refractive index of the solvent (we assume a protein medium with *n* = 1.4), the normalized emission of the donor in the absence of the acceptor (extracted from Figure S7 in the Supporting Information), and the decadic molar extinction coefficient of the acceptor, respectively. The ε*_A_*(λ) values of the acceptor as a function of λ, which are typically in units of M^–1^ cm^–1^, have been estimated using the Beer–Lambert law
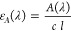
3where A (λ) is the absorbance as a function of λ, *c* is the concentration of the acceptor, and *l* is the length of cuvette. These values of *J*(λ) were employed to estimate the *R*_0_ values between donor and acceptors, as well as the *R*_0_ distance for homo-FRET, as listed in Table 1. The *R*_0_ values for energy transfer between F378/F555, F378/NR, and F555/NR are 8.2, 6.9, and 7.8 nm, respectively. For homo-FRET, the *R*_0_ distances for F378/F378, F555/F555, and NR/NR energy transfer is 5.5, 5.3, and 5.5 nm, respectively. Homo-FRET will not be detected by lifetime decay measurements and may thus occur in the investigated systems. However, we have not investigated homo-FRET quantitatively, and the rest of the discussion focuses on hetero-FRET, involving different types of molecules as donors and acceptors. The average distance between donor and acceptor molecules (*R*_DA_) can be calculated using the Förster radius *R*_0_ and efficiency of energy transfer for each pair. As shown in Table 1, the *R*_DA_ distance between F378 and F555 molecules in samples prepared by method A is 8.9 nm, while for samples prepared by method B the distance is 8.6 nm. In the case of F378 and NR, *R*_DA_ is 10.7 nm (method A) and 10.4 nm (method B). For the F555 and NR systems, *R*_DA_ is 8.8 nm (method A) and 8.3 nm (method B). All *R*_DA_ distances are in the range of 1–10 nm (in the case of the F378:NR donor acceptor pair, *R*_DA_ is just above 10 nm), typical of systems where non-radiative energy transfer between the two pairs will dominate over reabsorption processes.^59^

## 5. Application of Functionalized LPNFs

For applications of luminescent LPNF materials, it is desirable to convert the liquid LPNF dispersion into a solid form. We have investigated the conversion of the LPNF dispersion into thin films as well as into moldable gels.

**Figure 6:**
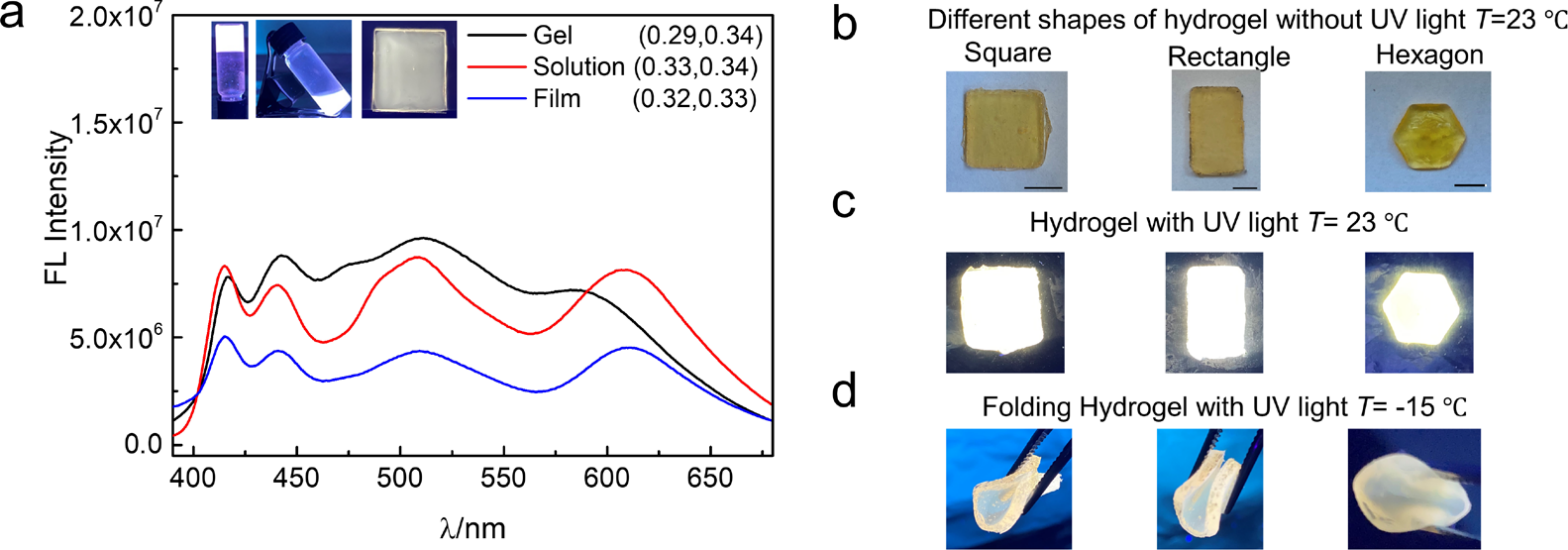
(a) Fluorescence spectra of different states of the white light-emitting matrix. Insets show photographs of white light emitting solution, spraying film (size, 2 × 2 cm), and gel under the UV light at 365 nm. (b) Hydrogel with different shapes under room temperature without UV light. Scale bar is 1 cm. (c) Hydrogel with different shapes under room temperature with UV light. (d) Hydrogel at a low temperature when pressed by a tweezer.

The liquid LPNF dispersion can be handled as an ink that can be directly sprayed onto substrates. In this process, the F378:F555:NR@PNF dispersion was transferred to a spray bottle. The dispersion could then be sprayed onto a glass substrate, resulting in an LPNF coating; SEM images of the sprayed film display a surface with fibrillar objects as outlined in Figure S1 (Supporting Information). When irradiated with UV light (365 nm), the F378:F555:NR@LPNF coating displayed emission of white light. Analysis of the emission spectrum (Figure 6a) results in CIE coordinates (0.32, 0.33), which is close to pure white.

The LPNF dispersions can also be turned into hydrogels by addition of a PVA: Gly binary solution. The F378:F555:NR@LPNF dispersion is mixed with a PVA solution (1:1, v/v), and the mixture is then kept at room temperature for 2 h, resulting in gels that under UV irradiation display emission of white light. Figure 6a shows spectra for both the starting liquid F378:F555:NR@LPNF dispersion and the F378:F555:NR@LPNF dispersion after formation of the gel (by addition of PVA: Gly). It should be noted that for the emission spectrum for the gel sample, the Nile red emission wavelength is blue-shifted for the systems involving PVA: Gly. A likely explanation is the lower polarity of this system.^60^ The initial aqueous LPNF dispersion yields CIE coordinates of (0.33, 0.34), whereas analysis of the emission spectrum of the gel yields CIE coordinates of (0.29, 0.34).

Moldable gels can be prepared by mixing the LPNF dispersion with the PVA: Gly binary solution. The gels can be cast simply by transferring the mixture into a suitable mold. Different shapes can thus readily be prepared such as square, rectangle, and hexagon (Figure 6b). Due to the blue shift of NR emission in the gels, it was necessary to increase the amount of NR to achieve white light, and as a result, the gels have an orange color when viewed under normal light. When exposed to UV light (365 nm), the gel emitted white light (Figure 6c). The gels are mechanically flexible, and the flexibility is retained even at low temperatures. Figure 6d illustrates the anti-freezing and mechanical performance of the gels, where the gels remain mechanically flexible even in a cold environment (around −15 °C). In addition, the temperature effect on the PL of spray-coated film and gels have been investigated (Figure S8, Supporting Information). The PL intensity of the gel and spray-coated films have been investigated as a function of temperature (by varying the temperature from 350 to 210 K). The luminescence increases with decreasing temperature, which is a typical result as non-radiative decay processes are suppressed at low temperatures.^61^

**Figure 7:**
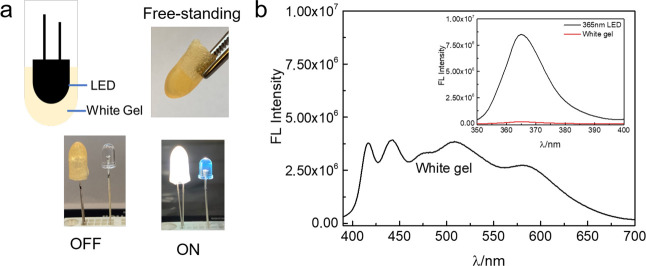
(a) Illustration formation of white gel coating on UV-LED (top) and white gel coating on UV LED (365 nm) with lights off (bottom left) and on (bottom right). (b) Fluorescence spectrum of white gel coating on UV LED (365 nm). Inset are photographs of relative absorbed intensity by white gel at wavelength of LED (365 nm).

The moldable gels can conveniently be used for coating of commercially available LEDs, thereby allowing tuning of the color of the emitted light. The LPNF:PVA:Gly mixture is put into a suitable mold, and the LED is then inserted into the sample. After 2 h, the LED can be taken out of the mold, resulting in a free-standing cover that can simply be put on top of a commercial LED (Figure 7a). We employed a commercially available UV-LED (365 nm), where the UV light was used as an excitation source for the F378:F555:NR@LPNF material. Figure 7b shows the spectra obtained when the UV-LED is in operation. As shown in the inset of Figure 7b, nearly all the photons from the UV-LED are absorbed by the gel. In addition, a commercial blue LED (440 nm) was coated with the same type of gel as described above, but with F555:NR@LPNFs. In addition, a film of F555:NR@LPNFs was spray-coated onto a glass slide, and the resulting sample was tested in remote mode at a distance of 2 cm from the LED. These experiments verified that white light can also be achieved in these systems involving a one-step FRET (Figure S9, Supporting Information).

**Figure 8:**
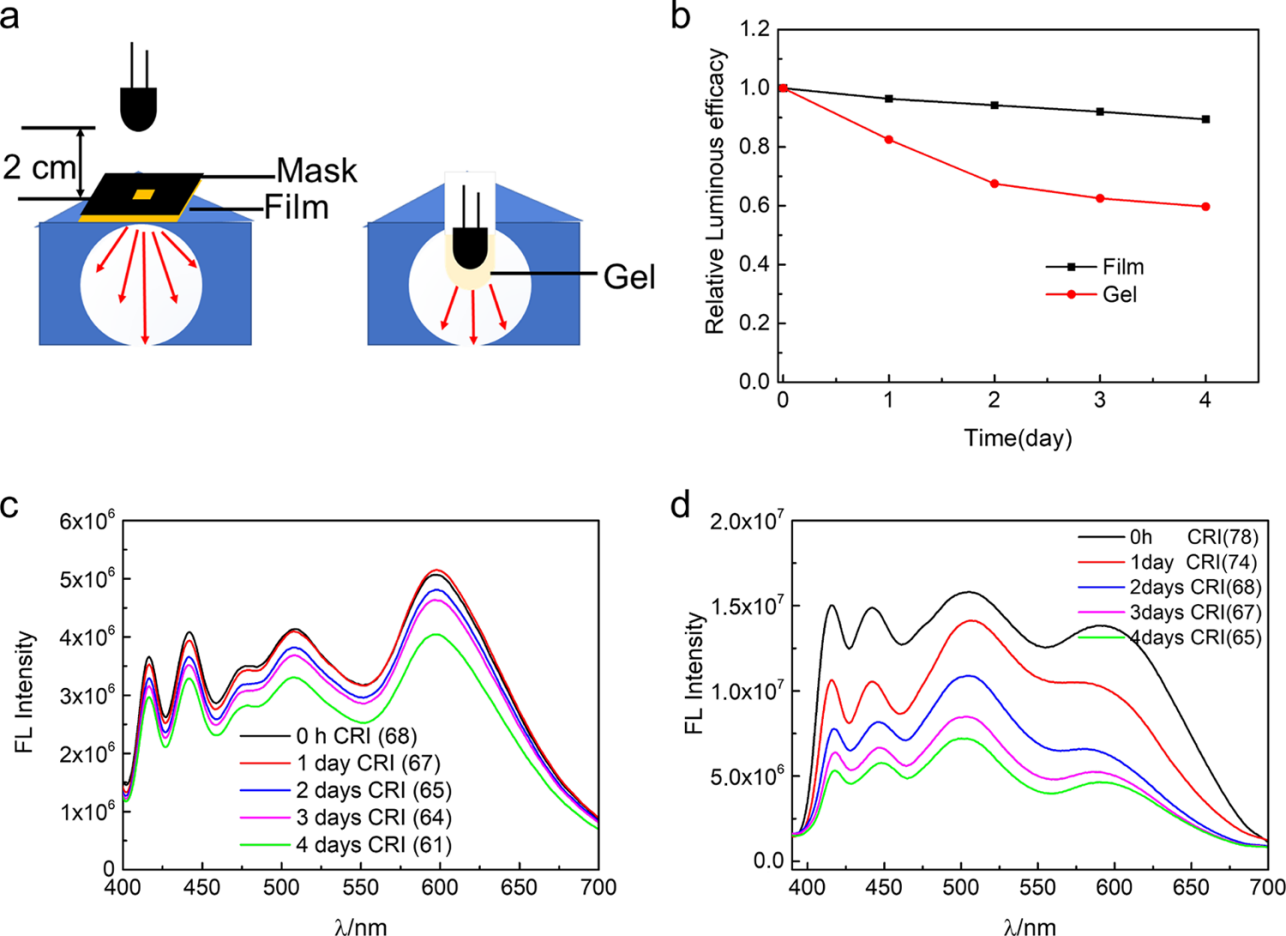
(a) Schematic illustration of the test of UV-pumped devices at a luminance of 91 cd/m^2^. (b) Relative changes in luminous efficacy of spray-coated films in remote mode and gel directly coated onto LEDs (in the case of the spray-coated film, the initial luminous efficacy is 28.1 lm·W^–1^; in the case of the gel, it is 47.5 lm·W^–1^). (c) Fluorescence spectra and CRI of spray-coated film in remote mode at a distance of 2 cm from the UV-LED (365 nm). (d) Fluorescence spectra and CRI of white gel directly coated on a UV-LED (365 nm).

In order to evaluate the suitability of the spray-coated films and gels as LED coatings, the stability and luminous efficacy were investigated as a function of time (Figure 8). The devices were operated at 3.5 V under ambient conditions for 4 days. The spray-coated film was operated in remote mode, whereas the gel was directly coated on top of the LED (Figure 8a). At the start of the measurements, both materials display excellent daylight emission, with a high color rendering index of 68 for the spray-coated film and 74 for the gel. The luminous efficacy was 28.1 lm·W^–1^ for the film and 47.5 lm·W^–1^ for the gel (Table S5, Supporting Information), while the correlated color temperature was 6111 K for the film and 7646 K for the gel (Table S5, Supporting Information). A shown in Figure 8b, after 4 days, there is a decrease in luminous efficacy of 40.7% for the gel and 10.7% for film (Figure S10, Supporting Information). The spectra obtained at different time points are shown in Figure 8c,d. A likely explanation for the decrease in luminous efficacy over time is photobleaching due to the presence of oxygen that can react with dyes in the excited state.^62^ Analysis of emission spectra were done in order to investigate the relative rate of degradation of the three dyes (Figure S11a,b, Supporting Information). It was found that F378 degraded faster than the other dyes in both the spray-coated film and the gel directly applied on the LED. However, the degradation process is slower in the case of the spray-coated film. Accordingly, for employment as LED materials, the spray-coated film operated in remote mode seems the most suitable, with a stability comparable to previously reported systems (Table S6, Supporting Information). In order to determine if temperature effects are involved in the case of the gel directly applied onto the LED, the temperature of the LED surface and the surface temperature of the gels were investigated with a thermographic camera (Figure S11c, Supporting Information). The results show that during LED operation, the surface temperature does not significantly change from the ambient temperature.

## 6. Conclusions

Many biopolymers are readily available structurally rich materials; however, for optical applications, it is often desirable to functionalize the biopolymer with luminescent dyes. Herein, we have demonstrated a novel approach to fabricate materials for conversion of UV light to white light. A protein capable of self-assembly into PNFs is ground with luminescent dyes and if mixed prior to self-assembly, nanofibrils are formed and are functionalized with multiple dyes. The dyes are statistically distributed in the nanofiber and undergo FRET processes, thereby enabling conversion of UV light to white light. On the other hand, if it is desirable to reduce the extent of FRET, PNFs can be formed from proteins ground with just one dye. If such PNFs functionalized with different dyes are mixed, FRET is less efficient. The described method is a convenient way to valorize protein materials enabling novel applications not suitable for the unmodified protein. The methodology should be readily extendable to proteins obtained from industrial waste streams and is thus a valuable addition to the toolbox for materials scientists enabling development of novel sustainable materials.^63,64^ We, moreover, demonstrate that the resulting functionalized PNFs can be converted into thin films by spraying or gels by addition of gelation agents. The gels can be molded into different shapes and can be used as phosphor coatings for inorganic UV-LEDs, enabling emission of white light. PNFs functionalized by multiple dyes are thus a readily processable versatile nanomaterial, available from low-cost protein sources.

## Supporting Information Available

Schematic illustration of spray coating and SEM images of films, absorbance spectra of different functionalized LPNF materials, fluorescence lifetime and quantum yield data, chromaticity coordinates, low-temperature PL, and stability test (PDF)
