# Supplementary material for: Mechanochemical Preparation and Self-Assembly of Protein:Dye Hybrids for White Luminescence
Source: ACS Appl Polym Mater. 2021 Sep 3;3(10):4825–36. doi: 10.1021/acsapm.1c00382 (PMC8506585; doi:10.1021/acsapm.1c00382)
Supplement: Supplementary file 1 — ap1c00382_si_001.pdf [file ap1c00382_si_001.pdf]

Supporting Information

Mechanochemical Preparation and Self-assembly of Protein: Dye Hybrids for White Luminescence

Yusheng Yuan, Niclas Solin*

Department of Physics, Chemistry, and Biology, Biomolecular and Organic Electronics, Linköping University, 581 83 Linköping, Sweden.

E-mail: niclas.solin@liu.se

1. Schematic illustration of dyes@LPNF composite films.

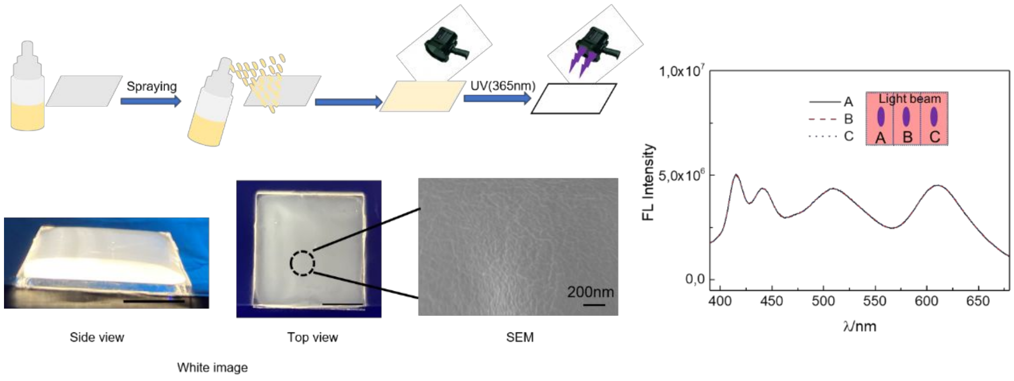

Figure S1. Schematic illustration of spray coating from the white matrix(top). Photographs of a spray coated white film from the side and the top, the scale bars represent 1 cm. The close-up is a SEM images of a spray coated white film. Left is the fluorescence spectrum of Film at different position.

2. Absorption spectra of F378@LPNF, F555@LPNF, NR@LPNF, F378@LPNF/F555@LPNF/NR@LPNF, and F378:F555:NR@LPNF.

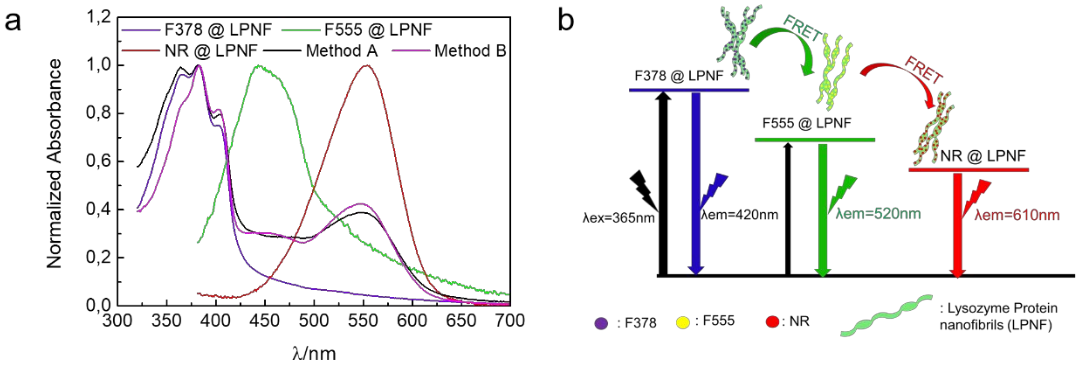

Figure S2. (a) Absorption spectrum of F378@LPNF (violet line), F555@LPNF (green line) and NR@LPNF (red line) separately, method A (F378@LPNF/ F555@LPNF / NR@LPNF, black line) method B (F378: F555: NR@LPNF, pink line). [F378]: 0.25 μg·mL^{-1}, [F555]: 0.25 μg·mL^{-1}, [NR]: 30 ng·mL^{-1}. (b) Schematic representation of FRET pathway in the matrix (F378→F555→NR). Excited states of F555 and NR could be populated by FRET and direct absorption.

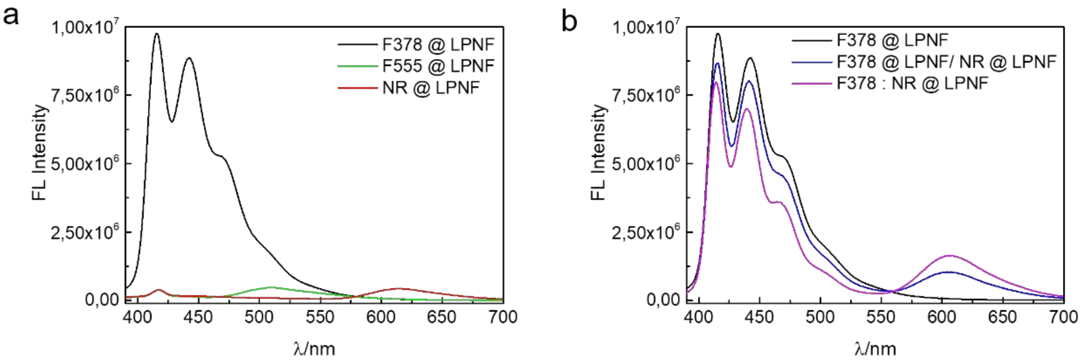

Figure S3. (a) LPNF functionalized with individual dyes excited at 365nm. (black line: F378@LPNF, green line: F555@LPNF, red line: NR@LPNF. (b) LPNF functionalized with F378 and NR dyes in different process excited at 365nm. black line: F378@LPNF, blue line: F378@LPNF/ NR@LPNF (Method A), pink line: F378:NR@LPNF (Method B).

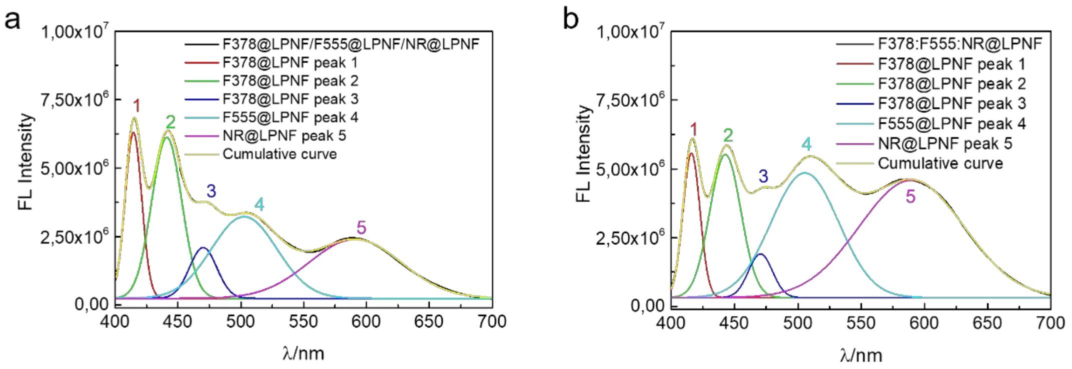

Figure S4. (a) deconvolution of fluorescence spectral data for a spectrum from a sample prepared by method A. (b) deconvolution of fluorescence spectral data for a spectrum from a sample prepared by method B.

3. Chromaticity Coordinates (Method B)

3.1 CIE values of F378: F555@LPNF systems in solution state.

Table S1. CIE Values of F378: F555@LPNF in solution state. The CIE Coordinates (X=0.20 and Y=0.28) Representing the ratio choosed for further investigate (Marked).

F378@HEWL ($\mu g \cdot mL^{-1}$)	F555@HEWL ($\mu g \cdot mL^{-1}$)	Chromaticity
0.25	0.125	(0.18,0.19)
0.25	0.25	(**0.20,0.28**)
0.25	0.375	(0.21,0.31)
0.25	0.5	(0.22,0.35)
0.25	0.625	(0.23,0.38)
0.25	0.75	(0.23,0.39)

Table S2. CIE Values of F555: NR@LPNF in solution state.

F555@HEWL ($\mu g \cdot mL^{-1}$)	NR@HEWL ($\mu g \cdot mL^{-1}$)	Chromaticity
0.25	0.125	(0.33,0.46)
0.25	0.25	(0.33,0.45)
0.25	0.375	(0.33,0.44)
0.25	0.5	(0.33,0.43)
0.25	0.625	(0.34,0.40)
0.25	0.75	(0.34,0.36)

Table S3. CIE Values of F378: F555: NR@LPNF in solution state. The CIE Coordinates (X=0.33 and Y=0.34) Representing the ratio for white emission (Marked).

F378@HEWL ($\mu g \cdot mL^{-1}$)	F555@HEWL ($\mu g \cdot mL^{-1}$)	NR@HEWL ($ng \cdot mL^{-1}$)	Chromaticity
0.25	0.25	5	(0.23,0.32)
0.25	0.25	10	(0.26,0.31)
0.25	0.25	15	(0.27,0.31)
0.25	0.25	20	(0.29,0.30)
0.25	0.25	25	(0.33,0.30)
0.25	0.25	30	(**0.33,0.34**)

4. Fluorescence lifetime measurements

Fluorescence decay profiles of individual dyes are monitored at its maximum emission wavelength upon excitation wavelength at 375nm, for the donor-acceptors are monitored at donor's maximum emission wavelength upon excitation wavelength at 375nm.

Table S4. Triple exponential fluorescence decay profiles of LPNF functionalized with dyes. F378@LPNF, F555@LPNF, NR@LPNF, Method A, Method B. [F378@LPNF]: 0.25 μg·mL^{-1}, [F555@LPNF]: 0.25 μg·mL^{-1}, [NR@LPNF]: 30 ng·mL^{-1}.

Samples	τ_1 (ns)	RW_1 [%]	τ_2 (ns)	RW_2 [%]	τ_3 (ns)	RW_3 [%]	τ (ns)	χ^2
F378@LPNF (monitored at 420nm)	0.13	4.13	0.75	78.62	1.83	17.25	0.91	1.15
F555@LPNF (monitored at 520nm)	1.01	7.77	4.93	43.08	9.36	49.15	6.8	1.21
NR@LPNF (monitored at 610nm)	0.32	5.31	1.02	35.55	2.44	59.14	1.82	1.12
F378@LPNF/F555@LPNF (monitored at 420nm)	0.4	38.73	0.52	57.11	2.58	4.16	0.56	1.27
F378: F555@LPNF (monitored at 420nm)	0.16	17.08	0.40	73.41	2.11	9.51	0.52	1.21
F378@LPNF/NR@LPNF (monitored at 420nm)	0.12	4.47	0.65	74.45	1.72	21.08	0.85	1.33
F378: NR@LPNF (monitored at 420nm)	0.47	13.69	0.65	67.80	1.82	18.51	0.84	1.29
F555@LPNF/NR@LPNF (monitored at 520nm)	1.31	14.72	3.94	57.33	7.78	27.95	4.63	1.20
F555: NR@LPNF (monitored at 520nm)	1.27	28.71	3.96	57.01	10.15	14.28	4.07	1.11
Method A Sol (monitored at 420nm)	0.07	2.47	0.60	76.69	1.79	20.84	0.83	1.22
Method B Sol (monitored at 420nm)	0.09	4.49	0.59	79.24	1.84	16.27	0.77	1.33
White gel (Method B, monitored at 420nm)	0.09	4.82	0.53	81.98	1.96	13.20	0.70	1.32
White film (Method B, (monitored at 420nm)	0.11	8.05	0.49	76.27	1.83	15.68	0.67	1.31

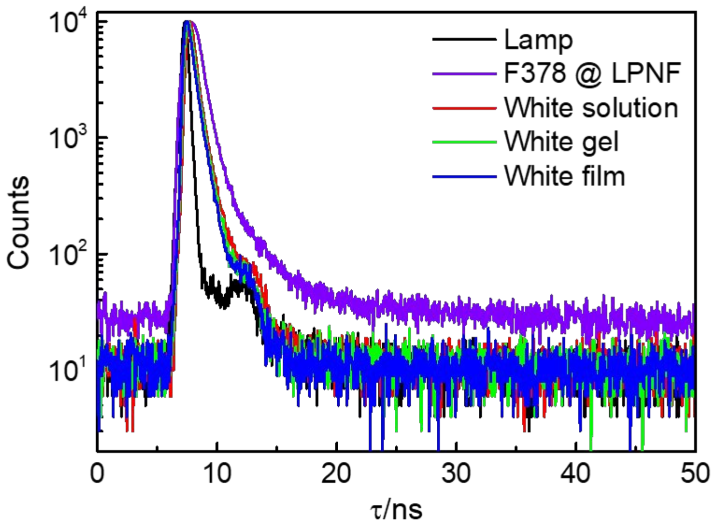

Figure S5. Fluorescence decay profiles of LPNF functionalized with dyes in different states prepared by method B. F378@LPNF (violet line, monitor at 420nm), Method B solution (red line, monitor at 420nm), Method B white gel (green line, monitor at 420nm), Method B sprayed film (blue line, monitor at 420nm). [F378@LPNF]: 0.25 μg·mL^{-1}, [F555@LPNF]: 0.25 μg·mL^{-1}, [NR@LPNF]: 30 ng·mL^{-1}.

5. Quantum yield of LPNF composites

The fluorescence quantum yield (Φ) of individual F378@LPNF, F555@LPNF solution samples were estimated by comparison with coumarin 153 in ethanol as standard (Φ = 0.53) [1], fluorescein in 0.1M NaOH as standard (Φ = 0.92) for NR@LPNF sample [2]. The quantum yield was calculated using the following equation [3]:

$$\Phi_{F,x} = \Phi_{F,s} \cdot \frac{Grad_x}{Grad_s} \cdot \frac{\eta_x^2}{\eta_s^2}$$

Where, subscript x denotes unknown sample and subscript s refers to standard. *Grad* is gradient from the plot of integrated fluorescence intensity vs absorbance, absorbance is at excitation wavelength and, η is the refractive index of the solvent used (0.1M NaOH is 1.33, Ethanol is 1.36, for PNF samples 1.42 is used).

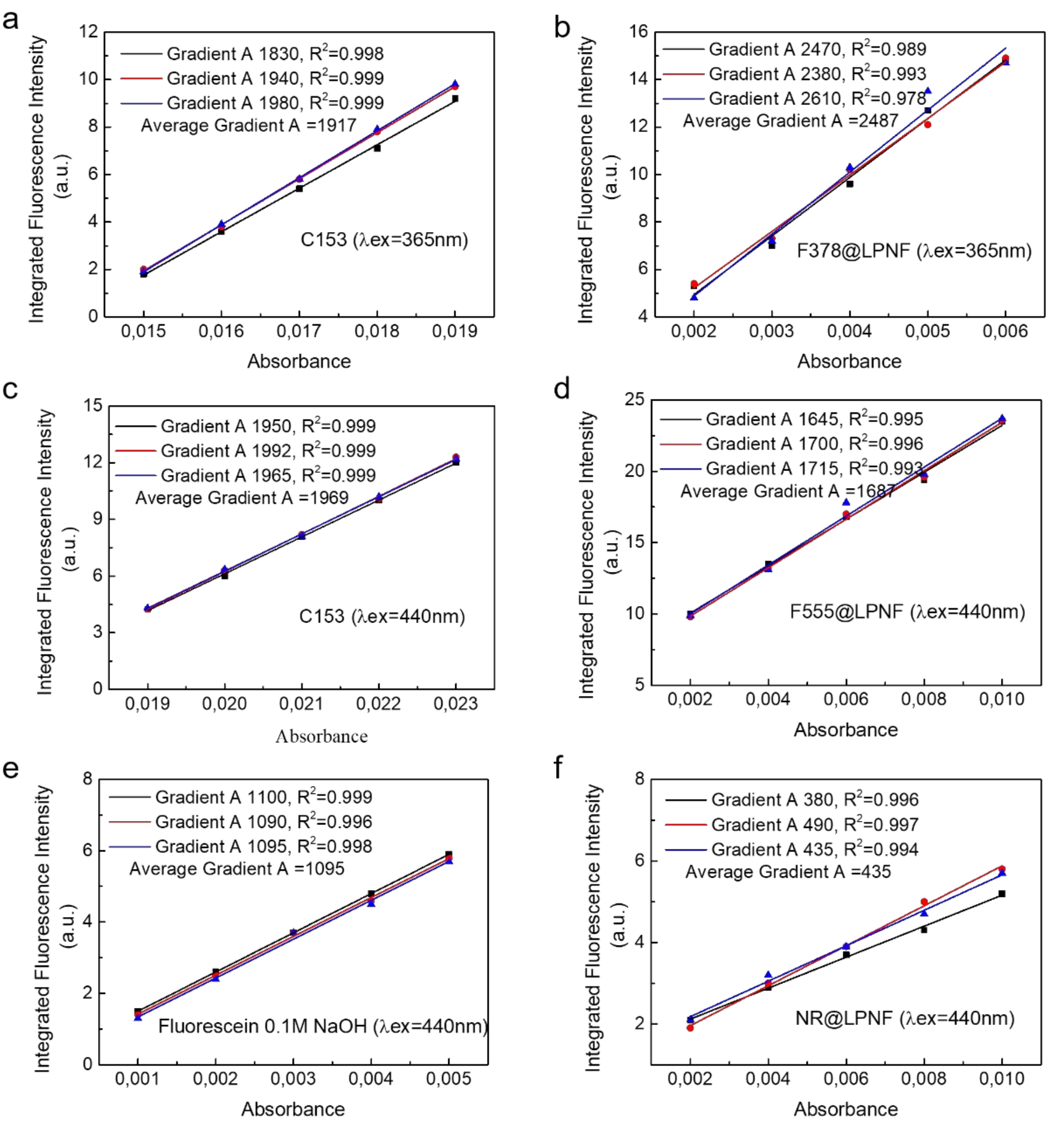

Figure S6. Linear plot of integrated fluorescence intensity vs absorbance. (a) C153 excited at 365nm. (b) F378@LPNF excited at 365nm. (c) C153 excited at 440nm. (d) F555@LPNF excited at 440nm. (e) Fluorescein excited at 365nm. (f) NR@LPNF excited at 440nm.

6. Spectral overlap of donors and acceptors

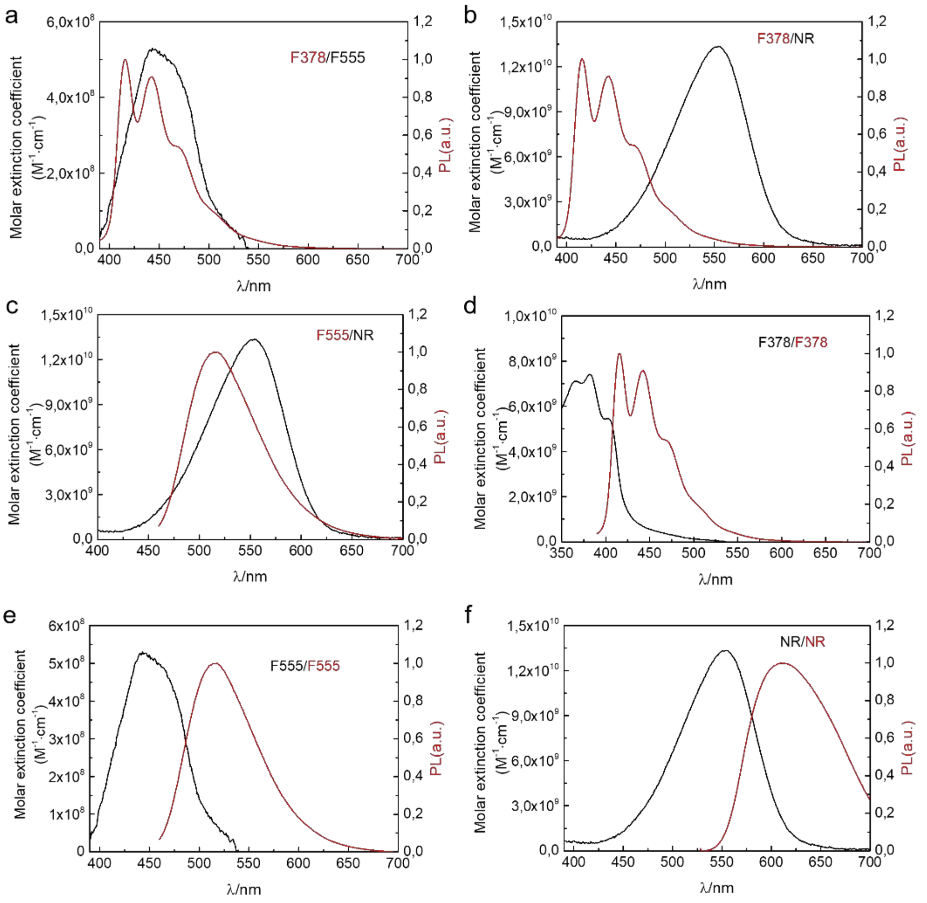

Figure S7. The spectral overlap between the absorption of acceptor (molar extinction coefficient, $M^{-1}\cdot cm^{-1}$) and the emission of donor for (a) F378/F555, (b) F378/NR, (c) F555/NR. (d) F378/F378, (e) F555/F555, (f) NR/NR. In all cases the the spectra are taken from the dye@PNF samples.

7. Temperature effect on the PL of spraying film and gel

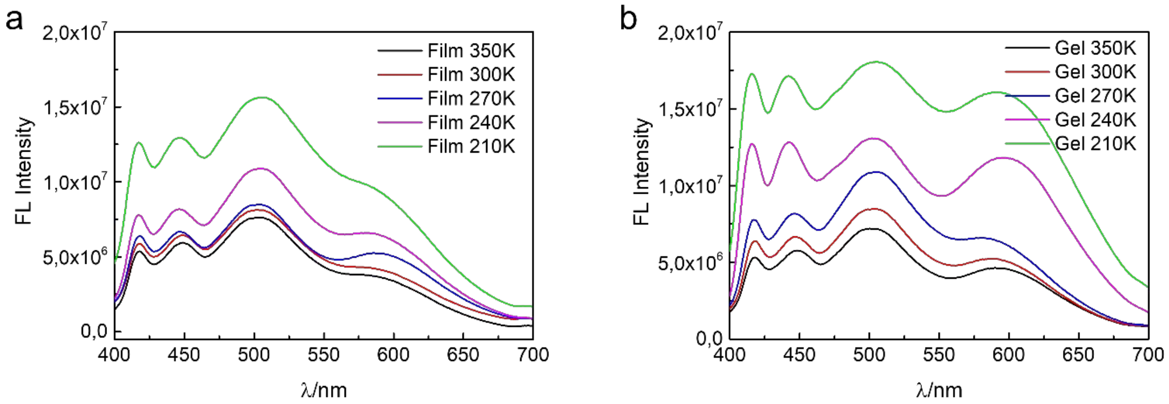

Figure S8. Temperature-dependent PL spectra of spraying film (a) and gel (b) measured at different temperatures from 210 to 350 K.

8. Commercial Blue LED coating with spraying film and gel

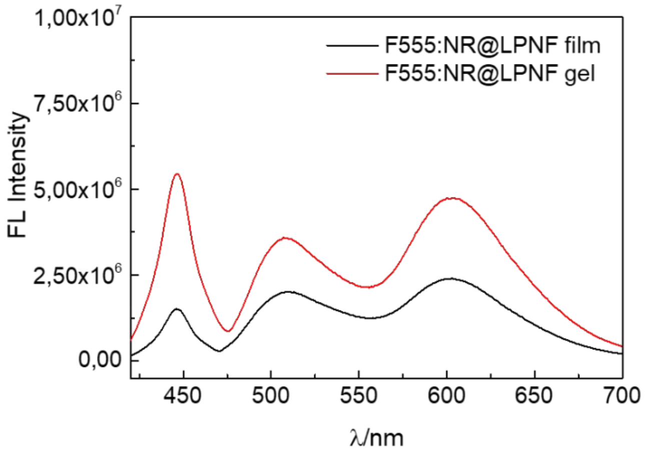

Figure S9. F555:NR@LPNF spraying film coating on the blue LED (440nm) at a distance of 2 cm. F555:NR@LPNF gel coating on the blue LED.

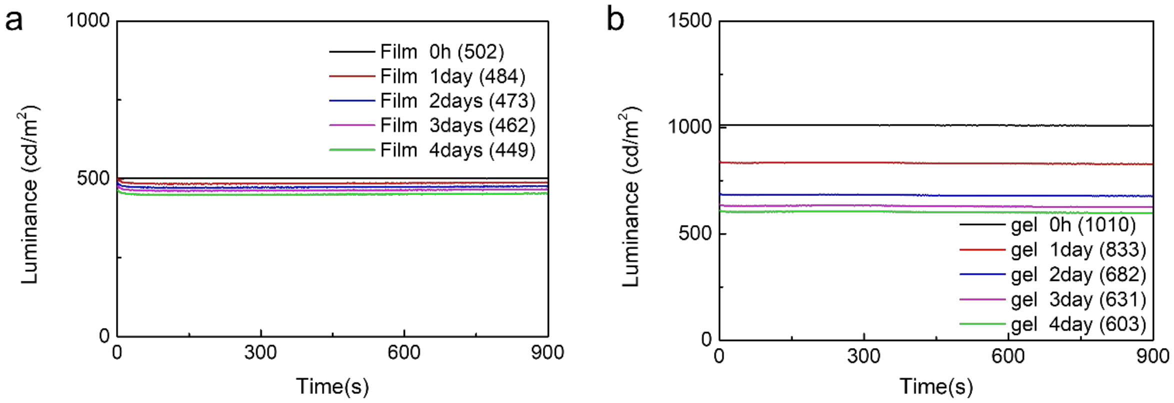

Figure S10. Luminance of white film (a) coating above the UV LED (365nm) at a distance of 2cm and white gel (b) coating on UV LED (365nm).

Table S5. Luminous efficacy and CCT of spraying film and gel on coating LED.

Time (day)	Film ($lm \cdot W^{-1}$)	Gel ($lm \cdot W^{-1}$)	Film CCT(K)	Gel CCT(K)
0	28.1	47.5	6111	7646
1	27.1	39.2	6221	8434
2	26.5	32.1	6467	8710
3	25.9	29.7	6544	8940
4	25.1	28.3	6782	9006

9. Stability test of spraying film and gel

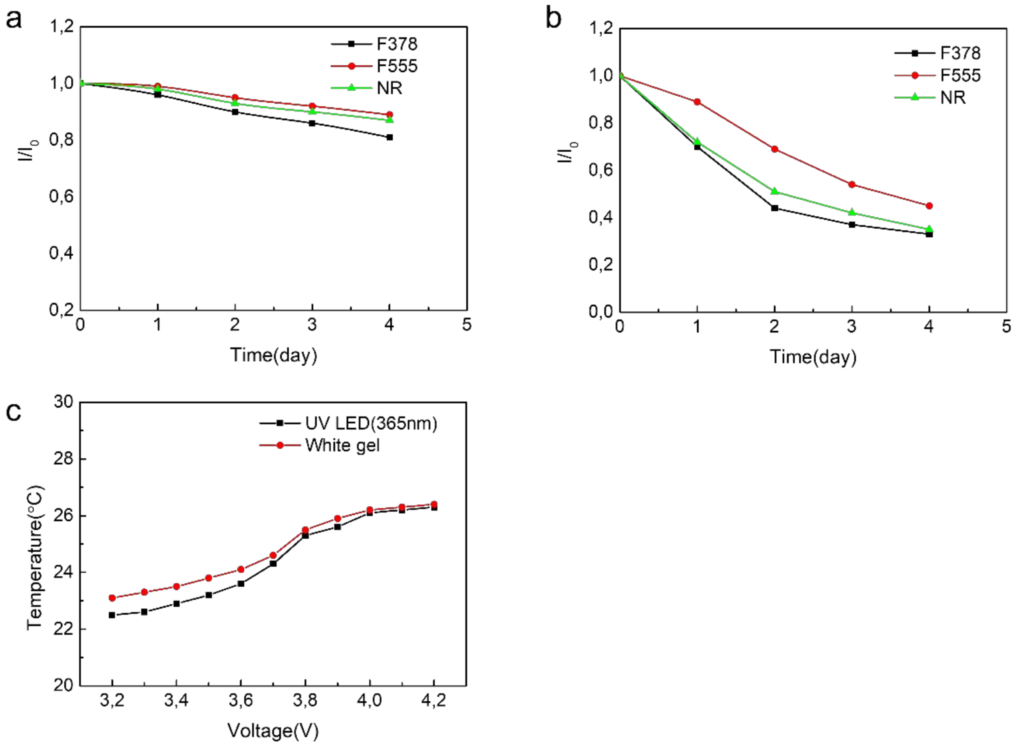

Figure S11. (a-b) Relative changes in intensity of emission spectra as a function of time during LED operation for (a) spray coated films and (b) gels. The emission intensity of each dye at a given time was divided by its emission intensity at 0 hours. F378 (black line, relative intensity at 420 nm); F555 (red line, relative intensity at 520 nm); NR (green line, relative intensity at 610 nm). (c) Changes in the temperature vs applied voltage for a commercial 365 nm LED (black) and the same LED with a white gel coating (red).

Table S6. Comparison with reported white light emitting devices on coating LED.

Materials	LED structure	Driving mode	CIE	CRI	CCT (K)	Stability	Conditions
Cellulose [4]	365LED/CNC /PGM/RG dyes	3V	-	84.4	3543-4150	3% CRI decrease, 24 hours.	Composite was deposited on a UV-LED
Protein-Au NCs [5]	380 LED blue/red AuNCs (prepared/measured in oxygen conditions)	30 mA	0.31/0.29	-	6840	50% efficiency decrease, 10 hours	Composite on a LED
BSA [6]	365 LED /BSA/C460/F/ ROX/EDC dyes	-	0.28/0.31	-	5300	50% luminance decrease, 106 hours	Biophosphors were placed at a fixed height (2.5mm) on LEDs in ambient air
Polyurethane [7]	460 LED/pm546/pm605/SRh101 dyes/MOFs	50 mA	0.47/0.41	85	2642	pm546: 70% luminance decrease, 24 hours pm605 and SRh101: 30% luminance decrease, 24hours	The phosphor was on the LED (phosphor fixed by highly transparent silicone).
This work	365 LED/RGB dyes	3.5V	0.32/0.33	68	6015	10.7% luminous efficacy decrease, 96 hours	Film on the LED (Remote)
This work	365 LED/RGB dyes	3.5V	0.29/0.34	78	7629	40.4% luminous efficacy decrease, 96 hours	Gel on the LED

References

(1) Würth, C.; Grabolle, M.; Pauli, J.; pieles, M.; Resch-Genger, U.; Relative and absolute determination of fluorescence quantum yields of transparent samples. *Nature protocols* **2013**, *8*, 1535-1550.

(2) Magde, D.; Wong, R.; Seybold, P. G.; Fluorescence quantum yields and their relation to lifetimes of rhodamine 6G and fluorescein in nine solvents: Improved absolute standards for quantum yields. *Photochem. Photobio*. **2002**, *75*, 327-334.

(3) Rurack, K.; Spieles, M.; Fluorescence quantum yields of a series of red and near-infrared dyes emitting at 600-1000 nm. *Anal. Chem*. **2011**, *83*, 1232-1242.

(4) Gotta, J.; Shalom, T. B.; Aslanoglou, S.; Cifuentes-Rius, A.; Voelcker, N. H.; Elnathan, R.; Shoseyov, O.; Richter, S.; Stable White Light-Emitting Biocomposite Films. *Adv. Funct. Mater*. **2018**, *28*, 1706967.

(5) Aires, A.; Fernández-Luna, V.; Fernández-Cestau, J.; Costa, R. D.; Cortajarena, A. L.; White-emitting Protein-Metal Nanocluster Phosphors for Highly Performing Biohybrid Light-Emitting Diodes. *Nano Lett*. **2020**, *20*, 2710-2716.

(6) Benson, K.; Ghimire, A.; Pattammattel, A.; Kumar, C. V.; Protein Biophosphors: Biodegradable, Multifunctional, Protein-Based Hydrogel for White Emission, Sensing, and pH Detection. *Adv. Funct. Mater*. **2017**, *27*, 1702955.

(7) Tang, Y., Cao, W., Yao, L., Cui, Y., Yu, Y., Qian, G., Polyurethane-coated luminescent dye@ MOF composites for highly-stable white LEDs. *J. Mater. Chem. C*. **2020**, *8*, 12308-12313.
